# Dual-Hemisphere Transcranial Direct Current Stimulation on Parietal Operculum Does Not Affect the Programming of Intra-limb Anticipatory Postural Adjustments

**DOI:** 10.3389/fphys.2021.789886

**Published:** 2021-12-20

**Authors:** Roberto Esposti, Silvia M. Marchese, Veronica Farinelli, Francesco Bolzoni, Paolo Cavallari

**Affiliations:** ^1^Human Physiology Section of the Department of Pathophysiology and Transplantation, Università degli Studi di Milano, Milan, Italy; ^2^Department of Biomedical Sciences, Humanitas University, Pieve Emanuele, Italy

**Keywords:** tDCS, parietal operculum, intra-limb APAs, voluntary movement, posture, human

## Abstract

Evidence shows that the postural and focal components within the voluntary motor command are functionally unique. In 2015, we reported that the supplementary motor area (SMA) processes Anticipatory Postural Adjustments (APAs) separately from the command to focal muscles, so we are still searching for a hierarchically higher area able to process both components. Among these, the parietal operculum (PO) seemed to be a good candidate, as it is a hub integrating both sensory and motor streams. However, in 2019, we reported that transcranial Direct Current Stimulation (tDCS), applied with an active electrode on the PO contralateral to the moving segment vs. a larger reference electrode on the opposite forehead, did not affect intra-limb APAs associated to brisk flexions of the index-finger. Nevertheless, literature reports that two active electrodes of opposite polarities, one on each PO (dual-hemisphere, dh-tDCS), elicit stronger effects than the “active vs. reference” arrangement. Thus, in the present study, the same intra-limb APAs were recorded before, during and after dh-tDCS on PO. Twenty right-handed subjects were tested, 10 for each polarity: anode on the left vs. cathode on the right, and vice versa. Again, dh-tDCS was ineffective on APA amplitude and timing, as well as on prime mover recruitment and index-finger kinematics. These results confirm the conclusion that PO does not take part in intra-limb APA control. Therefore, our search for an area in which the motor command to prime mover and postural muscles are still processed together will have to address other structures.

## Introduction

Keeping the balance of the whole body and of each of its segments during voluntary motor actions requires specific activities in postural muscles, which are called Anticipatory Postural Adjustments (APAs). APAs precede and counterbalance the postural perturbations induced by voluntary movements (Bouisset and Zattara, 1981; Bouisset and Do, 2008) and are programmed in a feed-forward way. Indeed, postural muscles activate prior to the onset of the focal movement, so as to develop one or more fixation chains toward the available support points. APAs are also involved in gait initiation, where their specific role is still debated: classic literature reports that they de-stabilize the body by displacing the center of mass (for a review, see Yiou and Caderby, 2017), while a recent study brings them back to the general role of body stabilization, as they appear to fix the trunk and upper body segments to the moving hip (Farinelli et al., 2021).

The role of APAs in whole-body stabilization is well-documented during movements involving large masses, like raising the arms or flexing a leg (Bouisset and Zattara, 1987; Breniere, 1987; Crenna and Frigo, 1991). These postural activities take the name of *inter-limb* APAs because they usually spread over several muscles of different limbs (Belen’kii et al., 1967; Friedli et al., 1984; Aruin and Latash, 1995). However, APAs are also produced when the voluntary movement involves only tiny masses, like flexing a finger or the wrist; in these cases, *intra-limb* APAs are observed in the same limb where the distal segment is moved (Aoki, 1991; Caronni and Cavallari, 2009a; see also Cavallari et al., 2016 for a review). These actions are needed in order to stabilize the proximal segments and to contribute to attain a higher precision of the focal movement (Hopf and Hufschmidt, 1963; Almeida et al., 1995; Bruttini et al., 2016).

Inter- and intra-limb APAs share several properties, like the ability to tune in to the needs of the postural context (Aruin and Latash, 1995; Hall et al., 2010; Bruttini et al., 2014), the adaptation to changes in movement speed (Esposti et al., 2015), and the important link with movement precision (Caronni et al., 2013; Bruttini et al., 2016). Moreover, several neural structures are involved in both inter- and intra-limb anticipatory postural control, like the primary motor cortex, the supplementary motor area (SMA), the sensorimotor areas, the pontomedullary reticular formation, and also subcortical structures such as the basal ganglia and the cerebellum (Viallet et al., 1987; Massion et al., 1999; Schepens and Drew, 2004; Schmitz et al., 2005; Caronni and Cavallari, 2009b; Petersen et al., 2009; Bolzoni et al., 2015, 2018; Bruttini et al., 2015; Farinelli et al., 2020).

With regard to the neural organization of voluntary movement and posture, an important finding suggests that the *postural* command governing APAs and the *focal* command that controls prime mover recruitment are functionally joined into a single *motor* command (Bruttini et al., 2014). However, transcranial Direct Current Stimulation (tDCS) applied on SMA separately modulated APAs without affecting the prime mover recruitment. This supports the idea that the *motor* command splits into its *postural* and *focal* components before entering SMA. It was therefore of interest to investigate hierarchically higher integration centers, maybe involved in supporting the APA adaptation to the postural context, thus being able to process both sensory and motor streams.

Recently, we investigated the role of Parietal Operculum (PO) because this neural structure is an important hub for integrating the proprioceptive and tactile information for the motor control (Milner et al., 2007; Sepulcre, 2014). Moreover, visual, somatosensory, and auditory functional streams, originating from different cerebral areas, converge in PO and are then headed to motor and premotor areas (Felleman and Van Essen, 1991). We started by using anodal or cathodal tDCS to selectively modulate the excitability of the PO contralateral to the moving segment (coPO, Marchese et al., 2019), while assessing intra-limb APAs associated to brisk flexions of the index finger, according to the paradigm of Caronni and Cavallari (2009a). Indeed, tDCS is known to modulate the cortical excitability by increasing or decreasing its activity, depending on the polarity. For example, anodal stimulation on the primary motor cortex transiently increases its excitability, while cathodal tDCS decreases it (Furubayashi et al., 2008; Tatemoto et al., 2013). TDCS also affects motor performance bilaterally, influencing finger sequence movements on both body sides (Vines et al., 2006). In fact, those Authors showed that anodal stimulation of the left motor area improved right-hand performance more than cathodal polarity, whereas opposite effects were induced on the left-hand. Against expectations, tDCS on coPO did not significantly affect the control of APAs associated to index-finger flexion: the intra-limb APA timing and pattern (which muscle showed excitation and which one inhibition) during and after tDCS of either polarity were at all comparable to those observed in the sham condition. These negative results suggested us that coPO may not be involved in the network governing APAs.

However, tDCS literature reports several cases in which the simultaneous stimulation of a given area in both hemispheres with opposite polarities (*dual-hemisphere*, dh-tDCS) elicited stronger effects than the unilateral tDCS. Given these premises, a new question opens: what will occur if the tDCS involves both POs? The dh-tDCS method interferes with the inter-hemispheric inhibition (Curtis, 1940), by which one hemisphere inhibits the contralateral one during the generation of voluntary unimanual movements (Boddington and Reynolds, 2017). This interhemispheric mechanism is revealed in healthy subjects by the inhibition of the contralateral motor cortex during movement initiation (see Beaulé et al., 2012, for a review); it was also proposed that interhemispheric inhibition would suppress mirror movements that may ruin the task performance, however it remains an unproven hypothesis.

Several Authors tried to exploit the inter-hemispheric balance in the cerebral network for increasing hand motor performance in healthy subjects and to recover motor function after brain injury (Fregni and Pascual-Leone, 2006; Williams et al., 2010). In this perspective, dh-tDCS has been proposed as a tool used to improve cerebral functionality. For example, Vines et al. (2008) tested the non-dominant hand and found that the simultaneous application of cathodal tDCS over the dominant motor cortex and anodal tDCS over the non-dominant motor cortex facilitated motor performance, with larger effects than the uni-hemisphere tDCS. Dh-tDCS has also been applied to stroke patients, trying to improve performance of the paretic hand by enhancing motor skill learning and reducing the spasticity (Lindenberg et al., 2010; Vandermeeren et al., 2012; Lefebvre et al., 2013; Vandermeeren and Lefebvre, 2015). However, the balance of results in the literature as well as the contribution of interhemispheric inhibition to motor deficits after stroke is still debated. Nevertheless, recent studies have shown that dh-tDCS improved cognitive functions (Cohen Kadosh et al., 2010; Kasahara et al., 2013) and tactile discrimination on the dominant hand of healthy subjects (Fujimoto et al., 2014, 2017). In particular, in Fujimoto et al. (2017) highlighted that dh-tDCS on PO was significantly more effective compared with uni-hemisphere tDCS.

Based on these findings, the aim of this study is to test whether the more powerful dh-tDCS set-up applied over PO could elicit significant effects on intra-limb APAs, which were not observed with unilateral tDCS. A positive result would suggest a PO involvement in APA neural control. Should also this protocol fail in affecting APAs and focal movement, we could definitely exclude such structure from the neural network governing APAs.

## Materials and Methods

Twenty subjects were enrolled in this two-groups, mixed-design, experimental study (13 males and seven females, mean age 26.7 ± 9.2 SD). Subjects were randomly assigned and equally distributed between the two stimulation polarities (see Neuronavigation and Tdcs for details). All volunteers were right-handed, as ascertained by the Oldfield handedness questionnaire. No subject had any history of neurological or orthopedic diseases, as well as of intake of drugs acting on the Central Nervous System. Participants provided their informed consent but were kept completely unaware of the stimulation condition. The experimental protocol complied with the policies and principles contained in the Declaration of Helsinki and were approved by the Ethical Committee of the University of Milan (counsel 6/19).

**Figure 1:**
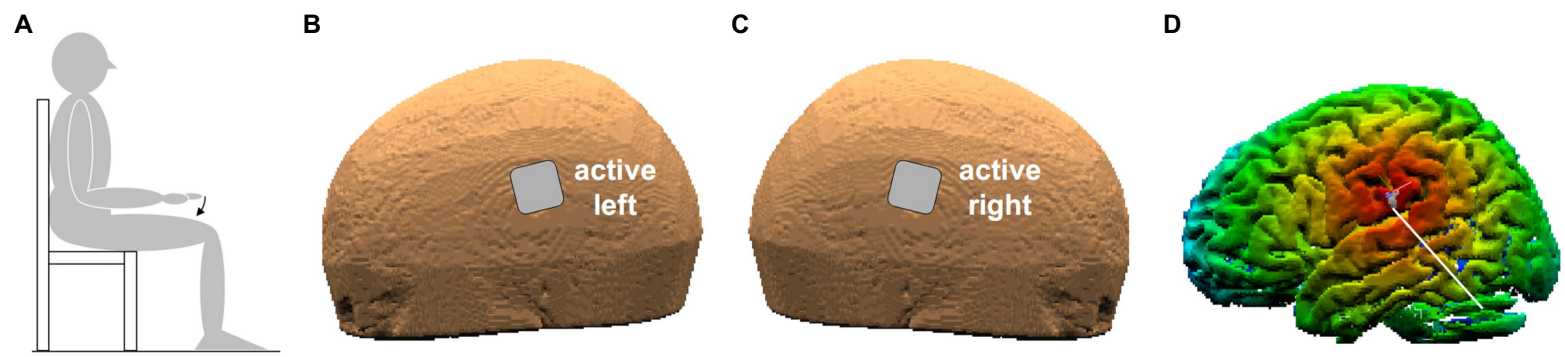
**(A)** Position of the subject. The arrow indicates index-finger flexion with the right hand prone. **(B,C)** Position of the two active electrodes (3.16 cm × 3.16 cm) on the left and right scalp. **(D)** False-color map of the distance between the tip of the neuronavigator pointing stylus, positioned on the left scalp, and the reconstructed brain surface. The white line points to the parietal operculum (PO) identified by means of its Talairach coordinates.

Subjects were sitting on a chair with both arms at rest. When performing the required movements (see below), the subject actively kept the right upper arm along the body with the elbow flexed at 90° and the hand prone, lined-up with the forearm. The index-finger was kept extended and aligned with the hand, while all other fingers were hanging. Throughout the experiment, subjects had to keep their back supported and both feet on the ground (Figure 1A). The experimenters supervised the subject’s position during the whole experimental session.

### Motor Task

All motor tasks were performed while recording EMG and finger movement (see Movement and EMG Recordings). First of all, the experimenter held the right upper limb of the subject, who was instructed to exert a Maximal Voluntary Contraction (MVC) of each of the recorded muscles, one at a time, for about 6–10 s. Then, the subject had to perform several sequences of 15 brisk flexion movements of the index-finger at the metacarpophalangeal joint: two sequences, with about 30 s of rest in between, were performed just before applying dh-tDCS (Pre), two at about half of the dh-tDCS period (Dur 10'), two in the last minutes of full-current dh-tDCS (Dur 20′) and two at 10, 20, and 30 min after dh-tDCS end (Post 10′, Post 20′, and Post 30′). Between each movement sequence, the subject rested his arm on a support. In order to avoid any reaction time, each movement was performed at will, after a beep (*ready* signal, repeated every 7 s). No subject complained about fatigue.

### Neuronavigation and tDCS

Transcranial direct-current stimulation was delivered by a neuroConn DC-Stimulator Plus (model 0021) connected to two sponge electrodes (3.16 × 3.16 cm), soaked with conductive gel. The two electrodes were placed on the scalp points closest to the PO of each side (Figures 1B,C), with either *ANODE LEFT* (cathode being on the right) or *ANODE RIGHT* polarity. The scalp positions were found by a neuronavigation system (SofTaxic Optic 2.0, see Figure 1D), on the basis of the average Talairach coordinates of the sub-areas PO1 and PO4, those closest to the subdural space, on the left (−52, −18.5, and 22) and on the right side (52, −18.5, and 22.5). Such values were taken from MNI coordinates in Eickhoff et al. (2006), converted to Talairach according to Lacadie et al. (2008). Both electrodes were fixed by elastic bands.

Dh-tDCS sessions started with a 60 s fade-in period, followed by 20 min DC at 2 mA and a 30 s fade-out. This current density (2 A/m^2^) was much lower than the safety limit (25.46 A/m^2^) reported on humans by Bikson et al. (2009) and even smaller than the minimal current density (142.9 A/m^2^, Liebetanz et al., 2009) that might induce brain lesion in the rat. No subject reported unpleasant sensations or could recognize the DC polarity. Throughout the experiment, it was checked that the scalp impedance was constant and never exceeded 5 kΩ (range 1.2–4.2 kΩ).

### Movement and EMG Recordings

The index-finger movement was recorded at the metacarpophalangeal joint on the right side by a strain-gauge goniometer (mod. F35, Biometrics Ltd.®, Newport, United Kingdom) stuck on the skin with hypoallergenic tape. Angular signal was DC amplified (P122, Grass Technologies®, West Warwick, RI, United States) and gain was calibrated before each experiment.

EMG signals were recorded by pairs of pre-gelled surface electrodes (H124SG, Kendall ARBO, Tyco Healthcare, Neustadt/Donau, Germany) placed on the prime mover Flexor Digitorum Superficialis (FDS) of the right upper limb and from the ipsilateral muscles Biceps Brachii, Triceps Brachii, and Anterior Deltoid (BB, TB, and AD, respectively) involved in stabilizing the arm (Caronni and Cavallari, 2009a). The inter-electrode distance was 24 mm and electrode placement followed the SENIAM guidelines (Hermens and Freriks, 1999). Recordings selectivity was verified by checking that activity from each recorded muscle, during its phasic contraction, was not contaminated by other muscular sources. EMG signals were amplified (IP511, Grass Technologies®, West Warwick, RI, United States) with a 1–20 k gain and a band-pass filter at 30–1000 Hz, so as to minimize movement artifacts and high frequency noise.

Conditioned goniometric and EMG analog signals were then sampled at 1 kHz, with an anti-aliasing low-pass filter at 500 Hz and a 12-bit resolution (A/D board model PCI-6024E, National Instruments®, Austin, TX, United States).

### Data Analysis

Each EMG recording was digitally rectified, and then the traces collected while moving the index-finger were expressed in % of the highest average EMG value recorded in a 1 s window during the subject’s MVC monitoring.

For each variable, the 30 traces recorded in the two sequences Pre tDCS were time-aligned to the point (trigger) in which finger flexion reached 15° with respect to its resting position (mean value from 1 to 0.1 s before the *ready* signal), and averaged. Such trigger choice actually granted the time-alignment precision, as it was verified that at 15° flexion, the index-finger was moving at more than 50% of its peak velocity. The resulting averaged trace extended from 2 s before to 0.3 s after the trigger. The same procedure was applied for the 30 traces obtained in Dur 10′, Dur 20′, Post 10′, Post 20′, and Post 30′. All subsequent measurements were taken on the averaged traces.

The onset of index-finger movement was automatically identified on the averaged goniometric trace. The mean signal level in the *reference period* from 1 to 0.5 s before the *ready* signal was subtracted from the trace, then an algorithm searched for the first moment in which the trace fell below −2 SD of the *reference period* and remained lower that that level for at least 50 ms. When the criterion was fulfilled, the algorithm searched backward the time point in which the trace started to deviate from the *reference period* mean value. All measurements were visually validated, in order to correct for possible failures of the automatic algorithm. Movement amplitude and duration were measured, respectively, as the amplitude and timing differences between the peak flexion of index-finger and the onset of its movement.

For each average EMG trace, the period from 1 to 0.5 s before movement onset was assumed as *reference*. The trace was integrated (time constant = 11 ms) and the mean *reference* level was subtracted from it; then the onset of an excitatory or inhibitory EMG change was identified by the above-described software algorithm, setting the threshold at +2 SD or − 2 SD of the *reference period* signal, respectively. The search was stopped at the onset of index-finger movement, so as to avoid any effect due to re-afferentation triggered by the focal movement. All timings were expressed as latencies with respect to FDS onset, with negative values representing time-advances. Finally, the amplitude of the EMG changes was measured as the mean level in the time-window from the onset of the EMG change to the onset of index-finger movement.

For each EMG and goniometric measurement, a two-way mixed-design ANOVA was applied to test for the effects of dh-tDCS *polarity* (ANODE LEFT vs. ANODE RIGHT, between-subjects factor) and *time* (Pre vs. Dur 10′ vs. Dur 20′ vs. Post 10′ vs. Post 20′ vs. Post 30′; within-subjects factor), as well as for their interaction. For all tests, statistical significance was set at *p* < 0.05; the effect size was expressed by the partial eta-square parameter (η^2^_p_). Power analysis regarded the *polarity* × *time* interaction (whether the within-subjects changes in *time* were different among *polarities*) and the main effect of *time* (whether changes occurred during or after dh-tDCS, independently from *polarity*), because these were the two meaningful effects to be evaluated. It was found that such ANOVA had 80% power to detect these two effects with an effect size as low as η^2^_p_ = 0.13, which in turn was half the effect size of the minimum significant difference we found when applying tDCS on SMA (Bolzoni et al., 2015).

## Results

**Figure 2:**
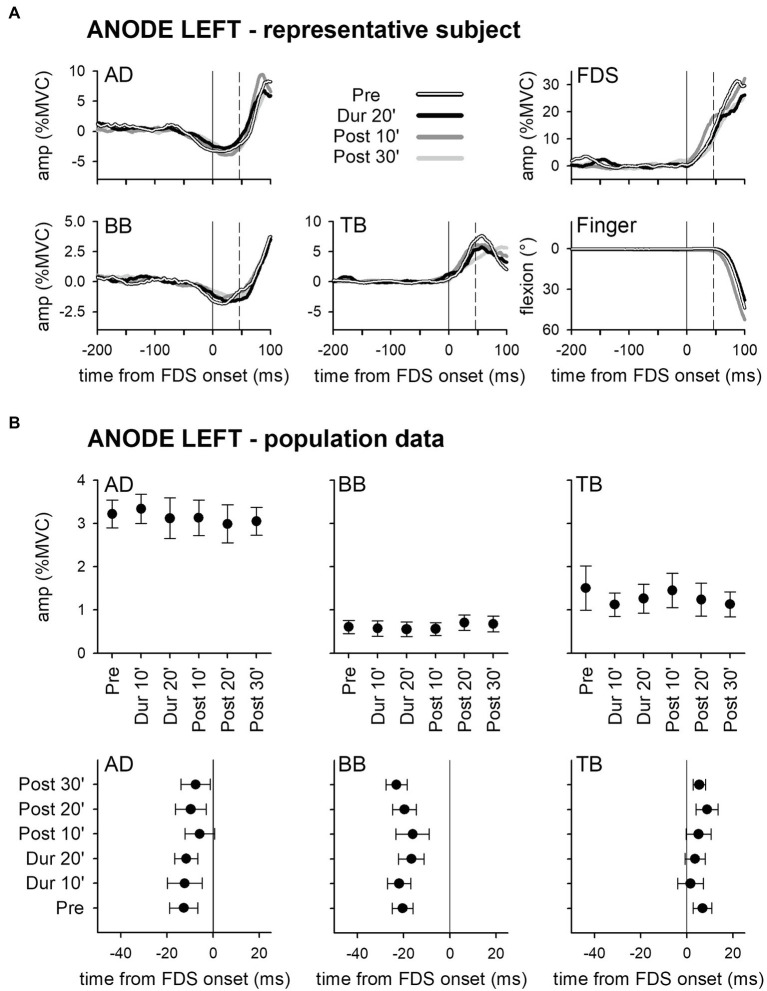
Rectified EMG and kinematics traces (shades of black in **A**) of a representative subject from the group who underwent dh-transcranial Direct Current Stimulation (tDCS) with the anode on the left PO and the cathode on the right one (ANODE LEFT). Averages of 30 movement trials, recorded immediately before dh-tDCS (Pre), in the last minutes of the full-current period (Dur 20′), and at 10 and at 30 min after it (Post 10′ and Post 30′). At all the time-points, the onset of activity (solid vertical line) in the prime mover Flexor Digitorum Superficialis (FDS) was accompanied by inhibitory Anticipatory Postural Adjustments (APAs) in Anterior Deltoid (AD) and Biceps Brachii (BB), and by an excitatory APA in Triceps Brachii (TB), which always preceded movement onset (dashed vertical line). Note how at each time point the traces are at all comparable, indicating that the DC stimulation had no effect on APAs, prime mover recruitment and focal movement kinematics. Population data of the ANODE LEFT group **(B)**. Amplitudes and latencies of APAs recorded in the AD, BB, and TB muscles are expressed as Mean ± SE. No significant changes occurred among the different time-points (Pre vs. Dur 10′ vs. Dur 20′ vs. Post 10′ vs. Post 20′ vs. Post 30′), confirming the stability of APAs.

**Figure 3:**
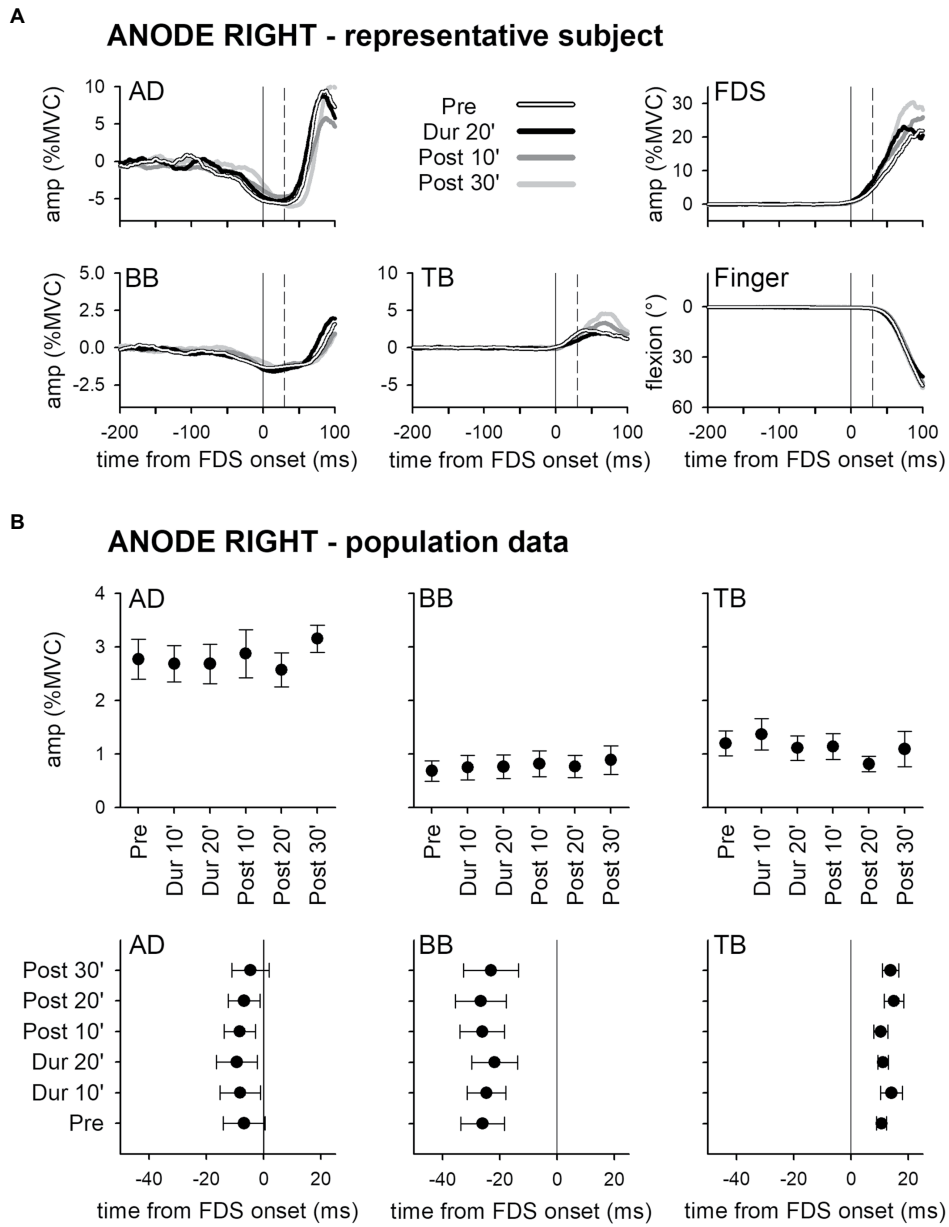
Traces of one representative subject **(A)** and population data **(B)** from the ANODE RIGHT group. Same layout as in Figure 2. It is apparent that also in this case DC stimulation had no effect on APAs.

Panel A of Figure 2 shows the mean traces of the integrated EMG and of finger kinematics signals recorded from a representative subject of the group who underwent dh-tDCS with the anode placed on the left PO and the cathode on the right (*ANODE LEFT*). Each trace is the average of the 30 movement trials that were recorded immediately before tDCS application (*Pre*), in the last minutes of full-current stimulation (*Dur 20′*), and after 10 and 30 min of recovery (*Post 10′* and *Post 30′*). Confirming Caronni and Cavallari (2009a), in *Pre*, the FDS onset (solid vertical line) was preceded by an inhibition in AD and BB, and by a burst of activity in TB. Notably, these EMG changes preceded movement onset (dashed vertical line) and stabilized the arm against the perturbation produced by the finger flexion; for these reasons, such actions are classified as APAs. All the traces recorded in *Dur 20'*, *Post 10'*, and *Post 30'* were comparable to those recorded in *Pre*. Figure 2B refers to population data and shows that the mean values of APA amplitude and latency obtained during and after *ANODE LEFT* dh-tDCS (including *Dur 10′ and Post 20′*) were at all comparable to those recorded in *Pre*. Experiments conducted in the subjects who underwent *ANODE RIGHT* stimulation (Figures 3A,B) led to results that actually replicated those obtained in the anode left group, confirming that either polarity had no effect on APA amplitude or latency.

These findings were also supported by statistics: two-way ANOVAs did not find any significant *interaction* (in all muscles, *F*_5,90_ ≤ 1.077, *p* ≥ 0.38, η^2^_p_ ≤ 0.056). The same was true for the main effects of *time* (*F*_5,90_ ≤ 1.713, *p* ≥ 0.14, η^2^_p_ ≤ 0.087) and *polarity* (*F*_1,18_ ≤ 2.657, *p* ≥ 0.12, η^2^_p_ ≤ 0.129). Finally, tDCS had no effect on amplitude of FDS recruitment and index-finger kinematics (two-way ANOVAs: *interaction F*_5,90_ ≤ 1.778, *p* ≥ 0.13, η^2^_p_ ≤ 0.090; *time F*_5,90_ ≤ 1.486, *p* ≥ 0.20, η^2^_p_ ≤ 0.076; and *polarity F*_1,18_ ≤ 1.506, *p* ≥ 0.24, η^2^_p_ ≤ 0.077).

## Discussion

The aim of this work was to re-test with a new approach whether the PO is engaged in the organization of intra-limb APAs. We addressed our studies to this structure in search of an area in which the postural and the voluntary components of the motor command, which split before entering SMA (Bolzoni et al., 2015), may be still processed together. We focused on PO as an important hub able to integrate multisensory streams within the motor organization (Milner et al., 2007; Sepulcre, 2014). Even though our recent work (Marchese et al., 2019) demonstrated that anodal or cathodal tDCS limited to the PO contralateral to the moving finger (coPO) did not disturb the APAs, here, we bilaterally stimulated both POs with opposite currents (dh-tDCS) as this approach has been proved to produce stronger effects than the unilateral stimulation in several neuromodulation studies. Dh-tDCS has been described to be a proficient tool to improve cerebral functionality (Vines et al., 2008) and to be more effective with respect to the uni-hemisphere approach. In particular, Fujimoto et al. (2017) reported that dh-tDCS over the POs, with the same 2 mA intensity used in the present study, actually produced a greater improvement in tactile orientation discrimination than the uni-hemisphere tDCS. Nevertheless, we did not observe any significant effect of dh-tDCS on amplitude or latency of intra-limb APAs associated with the index-finger flexion, in this way mimicking the results and confirming the conclusion of our previous work (Marchese et al., 2019).

Being confident, on the above premises, that dh-tDCS actually modulated PO excitability, it remains to exclude that our negative findings stem from experimental flaws. In this regard, the sample size seems adequate, as witnessed by the 80% power of ANOVA for the *time* effect and the *polarity* × *time* interaction; the two meaningful effects to be evaluated in our design (see Materials and Methods). It could be also argued that we did not perform sham dh-tDCS. However, Marchese et al. (2019) reported that sham on the coPO did not elicit any effect. Considering that in sham the 60 s fade-in period was immediately followed by the 30 s fade-out, so that current exposure was practically null, it is apparently unrealistic that repeating such protocol with the dual-hemisphere electrodes arrangement would lead to different results. The last important aspect is that in both Marchese et al. (2019) and in the present study, all finger flexions occurred with the arm kept in the same position (actively supported by the subject, with elbow at 90° and hand prone in axis with the forearm). It could then be debated that such experimental protocol does not allow to test the well-known ability of APAs to tune to the needs of the postural context in which the movement occurs (Aruin and Latash, 1995; Caronni and Cavallari, 2009a; Hall et al., 2010; Bruttini et al., 2014) and, consequently, it is unsuitable to highlight a possible involvement of PO in such function. However, the subjects had resting periods between each movement sequence and, in such periods; they laid their right arm on a support, thus implying a cyclic change between the *experimental* posture and the *resting* posture. Considering that dh-tDCS always started during a resting period, if PO were important for either perceiving the mechanical context or in integrating such information in the motor flow, one should expect a disruption of APA pattern or timing as soon as the subject assumed the experimental posture. Present results actually disproved such expectation.

On the other hand, the conclusion that PO is not involved in the control of APAs does not contrast with current literature. In fact, such area seems more influential on the earlier strategic phase of selecting the motor goal rather than in the planning phase of the motor act (Tunik et al., 2008; Woods et al., 2014; Valyear and Frey, 2015), while the commands for the prime mover and the related APA chains are defined in the latter phase. Moreover, the contribution of PO may concern more specific motor actions and learning-memory rather than the planning of motor acts. As evidenced by several works, PO network may be involved in other motor functions like working memory and tactile learning (Jäncke et al., 2001) and it might be more important for object-directed motor behavior (Maule et al., 2015). Additionally, the PO may modulate auditory-sensorimotor control, by mediating multimodal integration (Tanaka and Kirino, 2018), as well as being involved in orofacial muscles activities during phonation (Grabski et al., 2012).

On the bases of the present results and of the above considerations, we feel confident in concluding that our search for an area in which the motor command to prime mover and postural muscles are still functionally unique will have to address other structures, such as the premotor cortices.

## Data Availability Statement

The datasets generated for this study are available on reasonable request to the corresponding author.

## Ethics Statement

The studies involving human participants were reviewed and approved by Ethical Committee of the University of Milan (counsel 6/19). The patients/participants provided their written informed consent to participate in this study.

## Author Contributions

PC conceived the study and raised the funds. RE and SM conducted the experiments with the contribution of FB and VF and analyzed the results. PC, RE, and SM drafted the manuscript, which was approved, in the final version, by all authors. All authors contributed to the article and approved the submitted version.

## Funding

This study was supported by internal funds from “Laboratorio Analisi dell’Università degli Studi di Milano” (TARIFFARIO09_CAVALLARI).

## Conflict of Interest

The authors declare that the research was conducted in the absence of any commercial or financial relationships that could be construed as a potential conflict of interest.

## Publisher’s Note

All claims expressed in this article are solely those of the authors and do not necessarily represent those of their affiliated organizations, or those of the publisher, the editors and the reviewers. Any product that may be evaluated in this article, or claim that may be made by its manufacturer, is not guaranteed or endorsed by the publisher.
